# Supplementary material for: Chromothripsis during telomere crisis is independent of NHEJ, and consistent with a replicative origin
Source: Genome Res. 2019 May;29(5):737–49. doi: 10.1101/gr.240705.118 (PMC6499312; doi:10.1101/gr.240705.118)
Supplement: Supplemental Material [file supp_gr.240705.118_Supplemental_file_1.zip › contigs/annotated_contigs/DB50/contig.2.DB50_length_504_mean_cov_5.55158730159.docx]

**DB50_length_504_mean_cov_5.55158730159**

```
TATCCCAAGGCGGCTATTTCCAGTTCCCC|CCCCCGTCCCCCGCCATGTACACCAAAATTGGCAAGTTCTCAAGTACCTTATAAAAAAT
                              >chr20:45616610-45616857 - E=3e-123 p=0e+00
GGTAGAGTATTTGCATATAACCCACATACATCCTCCCATATACTTTAAATCATCTCTAGATTACTTATAATGTCCAACGCAATGTAAAC

ACTATGTAAACAGTTGTTATATGCATTGTTTTAAAACATATATTATTTTTATTCTGTATTTTTTTTTGCCCC|ATATATATATATATAT
                                                                         >chr20:48594792
ATATATATAT|GTACATATATATATATATATGTACAGCCCTTTATGTATTACAAATAGGAACCTCCACACTAACCTACACCTTGCTATA
-48595047 - E=7e-133
GTAACATTTTTTTGTTTTGAATCGTCATCACATGGGTAATGCTATAACAATATTCAGTGTTTTGAAAATCATTCCATATCATTACACGC

AGCTCCCTTAGTTTTCTGCAGGGATGCGGACTATTCATTGTATGGATTATAGCACAATCACA
```
